# Laboratory Research and Evaluation on Design and Application Performance of High-Performance Cold-Mix Resin

**DOI:** 10.3390/ma14174828

**Published:** 2021-08-25

**Authors:** Qing-Wei Zeng, Pei-Wei Gao, Yang-Fu Xu, Guo-Qing Dong, Man-Man Chen, Jing-Wei Zhao, Guang-Lai Jin

**Affiliations:** 1 College of Civil Aviation, Nanjing University of Aeronautics and Astronautics, Nanjing 211106, China; zqw@sinoroad.com (Q.-W.Z.); donggq@nuaa.edu.cn (G.-Q.D.); 2 Jiangxi Changtong Highway Co., Ltd., Nanchang 330025, China; xuyangfu2021@163.com; 3 Jiangsu Sinoroad Engineering Technology Research Institute, Nanjing 210000, China; cmm@sinoroad.com (M.-M.C.); jgl@sinoroad.com (G.-L.J.); 4 School of Transportation, Southeast University, Nanjing 211189, China; zaisong007@126.com

**Keywords:** epoxy resin, high-performance cold-mix resin, hot-mix epoxy asphalt, Marshall stability, fatigue performance

## Abstract

To improve the safety of orthotropic steel bridge decks and the construction efficiency of bridge deck pavement by enhancing the performance of pavement materials, a new-generation, high-performance cold-mix resin was prepared by carrying out the combination of micro-characteristic analysis and performance test. Meanwhile, the pavement performance and fatigue performance of high-performance cold-mix resin mixtures and hot-mix epoxy saphalt mixtures as a control group were studied experimentally. The results show that different kinds of epoxy resins show bisphenol structure in essence. The curing exothermic peak temperature of the cold-mix resin increases with the heating rate. Both the specific heat capacity (△*C*_P_) of cold-mix resin and cold-mix resin asphalts exhibit a sudden change between −20 °C and 40 °C. In resin asphalt mixtures, cold-mix resin forms the network structure skeleton whereas the asphalt distributed in the form of tiny particles. The dosage of respective component has a significant effect on the tensile strength and elongation at break of cold-mix resin. Compared with hot-mix epoxy asphalt mixtures, cold-mix resin mixtures exhibit comparable water stability and high and low-temperature performance, as well as greater fatigue life.

## 1. Introduction

Orthotropic steel bridges have advantages, such as being light, able to span large distances, a high bearing capacity and convenient construction [1,2,3,4]. At present, in the construction of long-span bridges (such as river and sea-crossing bridges) and overpass bridges (such as bridges over railways and highways), light-weight orthotropic steel bridge structures with the employment of epoxy asphalt as a paving material are more extensively used [5,6]. However, the large-scale construction of orthotropic steel bridges suffers certain disadvantages. The most prominent problems are fatigue damage of orthotropic plate structures and early damage to the bridge deck pavement [7,8,9] and the increasingly urgent difficulties in maintenance of steel bridge decks arising therefrom.

Nowadays, epoxy asphalt materials widely employed in China mainly includes hot-mix epoxy asphalt (HMA) and warm-mix epoxy asphalt (WMA). In order to obtain and maintain the fluidity of hot-mix epoxy asphalt during construction, a large amount of heat was supplied by machinery through energy consumption [10]. With the purpose of saving resources, research on the performance of warm-mix epoxy asphalt have aroused the interests of researchers; however, due to the low anti-rutting ability in the early stage and high cost, the application of warm-mix epoxy asphalt was limited [11,12]. Therefore, researchers paid more attention to improving the performance of epoxy asphalt mixtures. Tensile tests, rutting tests, Marshall tests was well as three-point beam bending tests were performed to research the EAC performance of epoxy asphalt (EA) and epoxy asphalt binder (ETB) with different proportions [13,14,15]. Fuhaid et al. [16] developed a kind of bio-based epoxy modified asphalt by using epoxy soybean oil (ESO) and bio curing agent maleic anhydride (MA), it was found that the corresponding mixtures showed higher Marshall stability than a mixture comprising the base asphalt. Through investigating the influence of polyethylene glycol on the mechanical properties of epoxy asphalt mixtures, Min [17] found that the incorporation of polyethylene glycol can significantly improve the low-temperature performance and toughness of epoxy asphalt mixtures. A novel approach of applying the combination of polyurethane and epoxy resin was proposed by Zhang et al. [18] to modify the asphalt mixtures, test results indicated that the optimal content of polyurethane and epoxy resin were 8 wt% and 32 wt%, respectively, the relevant asphalt mixtures exhibited superior low-temperature cracking resistance than epoxy asphalt mixtures.

Despite the fact that the incorporation of epoxy resin brought excellent performance for bridge deck paving [19,20,21], merely 0.75% content of epoxy resin can prepare asphalt mixtures with a great ability to resist creep deformation and spread load [22], the obvious stress concentration on the surface layer of the pavement greatly reduced the service life of the pavement. Moreover, excessive curing period brought challenges to the traffic. Therefore, novel paving materials were developed to improve the above-mentioned undesirable trends. High-performance cold-mix resin materials for steel bridge deck pavement can reduce the relative deflection of orthotropic plates and tensile strain of pavement surface by increasing the stiffness of orthotropic bridge deck systems [23,24], thus, improving the overall fatigue life of the steel bridge deck. With advantages including excellent workability, mechanical properties and weatherability, high-performance cold-mix resin materials are suitable for use as a complete maintenance system on steel deck pavements, including minor maintenance, preventive maintenance and in large and medium-scale maintenance. Specifically, they can be used in waterproof adhesive layers, concrete pavement layers, or sealing layers. It is one of the most effective ways to repair and reinforce damaged concrete beams or slabs with high-performance cold-mix epoxy asphalt mixtures [25]. Generally, cold-mix epoxy asphalt mixtures are pasted with adhesives over the part where tensile stresses of concrete beams and slabs occur and the direction of high-performance fiber-reinforced cold-mix epoxy asphalt mixtures is parallel to the direction of tensile stress. How to ensure the effective bonding between cold-mix epoxy asphalt mixtures and reinforced concrete [26,27], that is, cooperation of cold-mix epoxy asphalt mixtures and concrete, is one of key problems in repairing and reinforcement of beams and slabs. By using the similar method, steel beams can also be repaired and reinforced. In general, the bending strength and the flexural bearing capacity can be increased by 17% to 99% and by about 19% to 99%, respectively. As a cost-effective, environmentally friendly and functional pavement material, high performance cold-mix resin asphalt mixture has been widely studied by researchers. However, there are no universally standardized hybrid designs, acceptable material types, laboratory field-relevant performance tests and field-relevant performance indicators.

On the basis of the existing literature, this research aims to prepare a novel material with short curing time for bridge deck pavement. In this paper, three types of widely applied epoxy resins are tested by infrared (IR) spectrum to analyze specific components and the physical properties as well as the micro-characteristics of resin and resin asphalts are analyzed. By adjusting the proportion of respective component in resin, orthogonal tests of tensile fracture are designated and the optimal dosage of respective component is determined. Ultimately, a novel paving material is identified, namely, high-performance cold-mix resin mixtures, which exhibits equivalent pavement performance and superior fatigue performance compared with hot-mix epoxy asphalt mixtures.

## 2. Materials and Methods

### 2.1. Resin and Resin Asphalts

#### 2.1.1. Materials

Three typical epoxy resins provided by Nanjing Chengyou Resin Co. Ltd., Nanjing, China were prepared, including Dow resin, TY-RA resin and YC-YN resin. E-51 is a kind of epoxy resin with Bisphenol-A type structure, which is the preferred base materials for preparing cold-mix resins [28]; low-molecule polyamide resin 651 (also called polyaminamides) generated by polyamine and organic acid was used as modified amine curing agent; auxiliary materials including flexibilizer ETBN, diluter ETPEG, coupling agent KH-550 and accelerator DMP-30 were used for modification. A certain amount of E-51, 651, ETBN, ETPEG, KH-550 and DMP-30 were fully mixed and the mixture was cured at 60 °C for 16 h, then the high-performance cold-mix resin was made. In line with this operation procedure, the straight-run petroleum asphalt was mixed into mixtures and the high-performance cold-mix resin asphalts was also made.

#### 2.1.2. Infrared Spectrum

According to the test method proposed in GB/T 6040-2002 [29], the infrared spectrum analysis tests for three typical epoxy resins Dow, TY-RA and YC-YN were carried out on a Fourier Transform Infrared Spectrometer (FTIR) (Nanjing, China) [30]. The samples were prepared by using deposit-dip-spin-coating method. After preparing the samples with the *KBr* tablet method, the samples were dissolved using anhydrous ethanol solution and then coated onto *KBr* tablets. After the anhydrous ethanol solution was heated and evaporated, IR spectra of the coated materials were determined within the scanning range of 400 cm^−1^ to 4000 cm^−1^.

#### 2.1.3. Differential Scanning Calorimetry

The differential scanning calorimetry (DSC) (Nanjing, China) was used to measure the heat released from curing of high-performance cold-mix resin as well as the glass transition temperature of the cured high-performance cold-mix resin and high-performance cold-mix resin asphalts [31]. With the purpose of investigating the influence of heating rate on DSC curves of cold-mix resins, four heating rates, namely 5, 10, 15 and 20 °C/min were designated and the setup proceeded as follows. The flow rate of nitrogen in apparatus was kept at 25 mL/min, then fifteen milligrams of high-performance cold-mix resin sample was placed in to the test vessel immediately. Subsequently, the samples were heated from 60 °C to 200 °C at the rates of 5, 10, 15 and 20 °C/min, respectively. As for the measurement of glass transition temperature, fifteen milligrams of resin sample and resin asphalt sample were placed into the vessel, respectively. The vessels were then placed into the instrument and cleaned with nitrogen endured for 5 min before the heating operation. Then, the temperature was increased to 300 °C at a rate of 20 °C/min and decreased to the temperature 50 °C lower than the expected glass transition temperature at the same rate. After that, the temperature was increased to 300 °C at a rate of 20 °C/min and the glass transition temperature of aforementioned two materials was measured.

#### 2.1.4. Microscopic Appearance

Scanning electron microscopy (SEM) (Nanjing, China) is an observation method somewhere between transmission electron microscopy (TEM) and optical microscopy. The resolution of the SEM used in this paper reaches 1 nm and the microscope can be continuously adjusted at the magnification of 300,000 times and above. The cured high-performance cold-mix resin samples were brittle fractured in liquid nitrogen, then tetrahydrofuran (THF) was added and heated together at 65 °C for 15 h. After the samples were dried at room temperature, the cross-section of the samples were sprayed with gold, then the samples were placed in the fixture of the instrument to observe the microscopic appearance of the cross-section. In order to better observe the microscopic appearance of high-performance cold-mix resin mixed with asphalts, the corresponding test was carried out on a laser scanning confocal microscope (LSCM) (Nanjing, China) [32]. After the resin and asphalt were evenly mixed, a drop of sample solution was dropped on the glass slide and then placed in an oven cured at 120 °C for 4 h. The samples were placed into the apparatus and fixed, then the 488 nm blue laser irradiation was applied and different materials in resin asphalts refract different characteristics under the emitted light.

#### 2.1.5. Fracture Tensile Test

The fracture tensile test is regarded as an important method to evaluate the basic mechanical properties of high-performance cold-mix resin. In accordance with the designed orthogonal experiments, a certain amount of E-51, 651, ETBN, ETPEG, KH-550 and DMP-30 were mixed and stirred more than 180 s in each set of tests. The mixtures were poured into the mold and then placed in an environmental box cured at 25 °C for 12 h. Afterwards, the samples were cured at 60 °C in an oven for 16 h, then fracture tensile tests were carried out on QJ211S (Nanjing, China) test machine to measure the tensile strength and percentage of breaking elongation of high-performance cold-mix resin. The tensile rate in test was set at 10 mm/min.

### 2.2. High-Performance Cold-Mix Resin Mixtures and Hot-Mix Epoxy Asphalt Mixtures

#### 2.2.1. Mix Proportion

Through the orthogonal experiment designed in fracture tensile test and further analysis, the best ratio indicates that the copies of E-51, 651, ETBN, ETPEG, KH-550 and DMP-30 in high-performance cold-mix resin were 50, 55, 50, 6, 3 and 2, respectively. The coarse and fine aggregates of the same type as those applied in epoxy asphalt concrete pavement of steel bridge deck were selected. The coarse and fine aggregates as well as mineral powder were rolled from basalt and limestone, respectively. In addition, polyester staple fiber accounting for 0.1% of the mass of mineral raw materials was also employed. Table 1 shows the grading range and composite gradation of high-performance cold-mix resin mixtures. The optimal asphalt-aggregate ratio was 7.5% and the corresponding porosity, Marshall stability and flow value were 1.6%, 80 kN and 2.86 mm, respectively.

Meanwhile, hot-mix epoxy asphalt mixtures were designed for comparison with high-performance cold-mix resin mixtures. The coarse and fine aggregates applied in hot-mix epoxy asphalt mixtures are the same as those applied in high-performance cold-mix resin mixtures. The hot-mix epoxy asphalt mixtures were prepared in accordance with the following procedures: the modifier, curing agent and base asphalt were heated to 80 °C, 60 °C and 170 °C, respectively, then the modifier and curing agent were mixed according to the weight ratio of 1:2. The obtained mixtures were mixed and stirred with epoxy resin in a 1:1 weight ratio, followed by the addition of aggregates which were heated to 185 °C. Table 2 shows the grading range and composite gradation of hot-mix epoxy asphalt mixtures.

#### 2.2.2. Pavement Performance Test

As a significant index to evaluate the pavement performance of road materials, water stability is mainly applied to measure the deterioration of the performance of mixtures under hydrodynamic pressure. In this section, the widely applied water immersion Marshall test and freeze-thaw splitting test were performed on high-performance cold-mix resin mixtures and hot-mix epoxy asphalt mixtures by following the standard test procedure in in JTG E20-2011 [33] and the water stability of each mixture was comprehensively evaluated by two indexes, namely residual Marshall stability and freeze-thaw splitting tensile strength ratio. Corresponding to each kind of pavement materials, a total of sixteen standard Marshall specimens were cast for water stability tests. Both water immersion Marshall test and freeze-thaw splitting test consisted of two groups of specimens, with each group including four specimens. In water immersion Marshall tests, two groups of specimens were placed in water at 60 °C for 0.5 h and 48 h, respectively. Afterwards, the Marshall stability of all specimens were measured. In freeze-thaw splitting tests, one group of specimens was placed in atmosphere whereas the other group of specimens was treated with saturated water for 15 min first, then placed in water for 0.5 h and cured for 16 h at −18 °C, followed by placing in a water tank with constant temperature of 60 °C for 24 h. Afterwards, two groups of specimens were all placed in water at 25 °C for 2 h and then respective splitting strength was measured.

Under the combined effect of high temperature ambient and vehicle load, the road materials would soften and then deform. In addition, the combined action of low temperature ambient and vehicle load would cause pavement cracking and finally, induce the structural damage under adverse environmental conditions such as rain and snow erosion. Therefore, the high and low stabilities of the designed mixtures is also an important part of the road performance research. In view of this, the rutting test and beam bending test confirming to the standard test procedure in JTG E20-2011 [33] were carried out to study the high- and low-temperature performances of target mixtures. Corresponding to each kind of pavement materials, four standard rutting specimens with the size of 300 mm × 300 mm × 50 mm were prepared. Three rutting specimens were used for high temperature performance tests and four prismatic specimens with the size of 250 mm × 30 mm × 35 mm cut from the remaining one rutting specimens were prepared for low temperature performance tests. The temperature rutting tests was 60 °C, the pressure generated by rubber tire was 0.7 MPa and the loading rate was 42 cycles/min. In beam bending tests, the temperature was −10 °C and the loading rate was set 50 mm/min.

#### 2.2.3. Four-Point Bending Fatigue Test

The four-point bending fatigue tests referred to the method suggested in JTG E20-2011 [33]. Corresponding to each kind of pavement materials, a standard plate with the size of 400 mm × 300 mm × 75 mm was formed by wheel and then cut into four beam specimens with the length of 380 mm ± 5 mm, width of 63.5 mm ± 5 mm and thickness of 50 mm ± 5 mm. After being cured for 4 h in an environmental chamber at 15 °C, the beam specimens were put into the loading device and fixed with clamps. Subsequently, the strain controlled sinusoidal waves were applied at a frequency of 10 Hz, then test began. The termination of the fatigue test was marked by the decrease of bending stiffness modulus to 50% of the initial bending stiffness modulus.

## 3. Results and Discussions

### 3.1. IR Spectra

**Figure 1:**
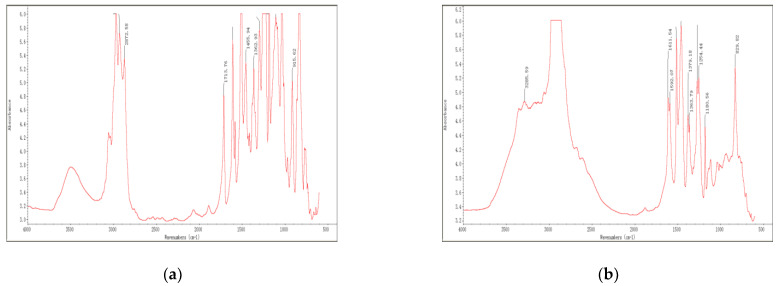
IR spectra of Dow epoxy resin: (**a**) component A; (**b**) component B.

**Figure 2:**
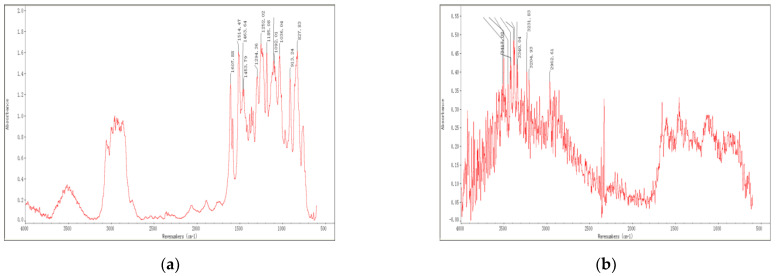
IR spectra of TY−RA epoxy resin: (**a**) component A; (**b**) component B.

**Figure 3:**
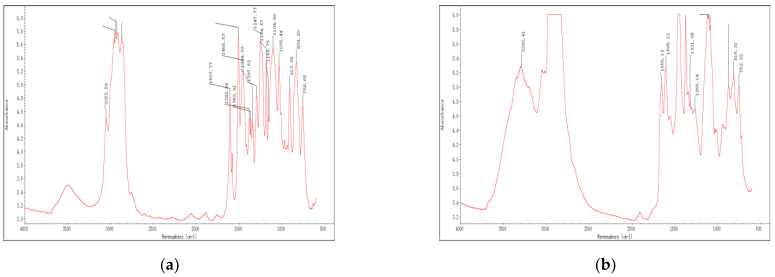
IR spectra of YC–YN epoxy resin: (**a**) component A; (**b**) component B.

The IR spectrum of components A and B in three typical epoxy resins, namely Dow epoxy resin, TY-RA epoxy resin and YC-YN epoxy resin, are shown in Figure 1, Figure 2 and Figure 3, respectively. It can be observed from FT-IR images obtained through IR spectrum analysis that, epoxy resins exhibit the characteristic peaks of epoxy groups [34]. The main absorption peak is located at 912 cm^−1^ and the vibration absorption peak of the benzene ring skeleton in the bisphenol-A structure is found at 1607 cm^−1^. Some epoxy resins are plasticized, toughened and modified by mixing with asphalt. The broad band at 3285 cm^−1^ shows the vibration absorption peak of hydroxyl groups or the double-frequency absorption induced by stretching vibration of N-H and carbonyl. There are many impurities represented by peaks between 900 cm^−1^ and 1300 cm^−1^ in the fingerprint region.

A comparison with the data from spectrum library shows that Dow epoxy resin is very similar to bisphenol-A resin in terms of component A, both mainly containing DER332. Such resin show epoxy equivalent of 172 to 176 (g/eq) and an epoxy value of 0.56 to 0.58; and the main ingredient of component A in TY-RA epoxy resin is DER 324, as well as doped asphalts, whereas the main ingredient of component A in YC-YN epoxy resin is EPON 828 as well as doped asphalts. Component B in epoxy resins mainly includes various curing agents and curing of epoxy resins at room temperature generally relies on an amine curing agent, in particular, polyamides. Based on analysis of IR absorption spectra, the component B in Dow epoxy resin shows an obvious band near 3000 cm^−1^ to 3100 cm^−1^, representing the vibration absorption peak of hydroxyl. By comparing the results, the peak is found to be ascribed to Hardener XU-HY 943, mainly including (N, N) dimethyldipropylamines and polyamides. The component B in TY-RA epoxy resin presents characteristic peaks of polyamides, namely, strong peaks at 1640 cm^−1^ and 1550 cm^−1^. The comparison demonstrate that the peaks are mainly attributed to Paramul ERO-SB compound, which mainly comprises polyamides. YC-YN epoxy resin are similar to TY-RA epoxy resin in component B, which is a room-temperature curing agent containing polyamides.

Based on the above analysis, the results of IR spectrum analysis of three typical epoxy resins are list in Table 3. The molecular structure and group characteristics of the typical epoxy resin materials were analyzed through their IR spectra. The result reveals that epoxy resins with bisphenol structures are commonly used and can be plasticized, toughened and modified by adding some asphalts. Component B mainly serves as the polyamide curing agent generally for curing at room temperature. It can be plasticized, toughened and modified by adding part of rubber and asphalt and colored by adding a little carbon black to ensure the desired color of roads.

### 3.2. DSC

**Figure 4:**
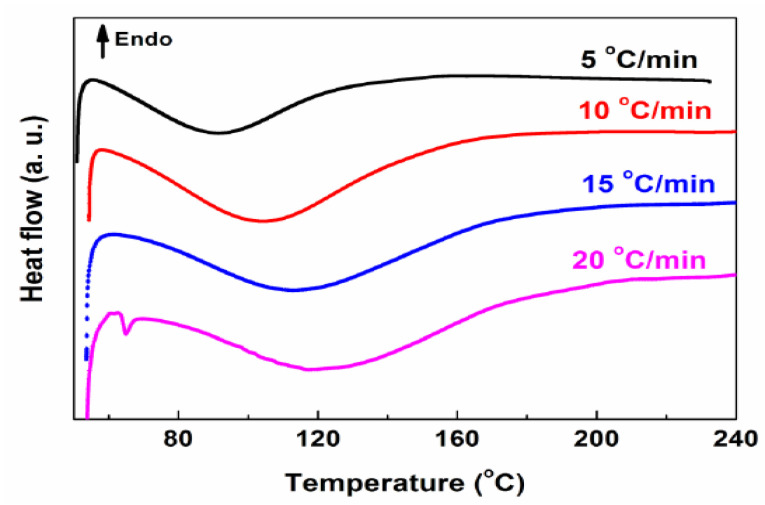
DSC curves of high-performance cold-mix resin at different heating rates.

Figure 4 shows the curing heat of high-performance cold-mix resin at different heating rates. As illustrated in Figure 4, after the curing reaction between the epoxy group in epoxy resin and the curing agent, different exothermic peaks appear at different heating rates of 5, 10, 15 and 20 °C/min and the faster the temperature rises, the higher the peak temperature, namely 90, 105, 115 and 125 °C.

**Figure 5:**
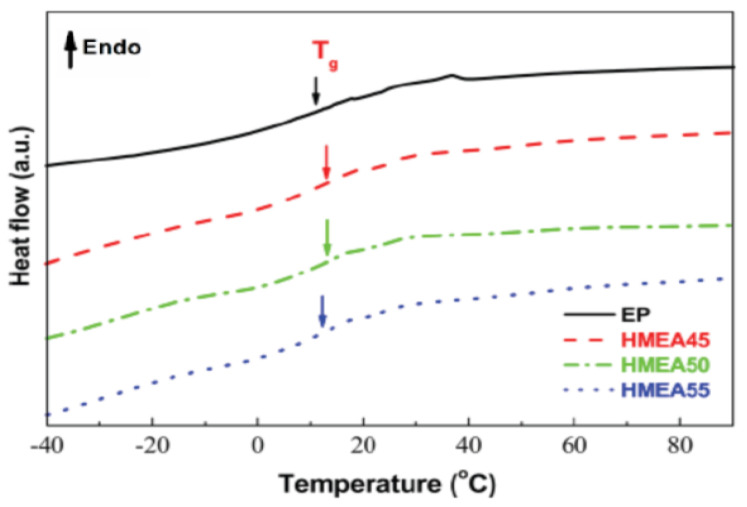
Glass transition temperature of the cured high-performance cold−mix resin and cold−mix resin asphalts.

The glass transition temperature (*T*_g_) plays a significant role in the complex low-temperature behavior of materials [35]. Figure 5 shows the glass transition temperature (*T*_g_) of completely cured high-performance cold-mix resin and high-performance cold-mix resin asphalts. It can be intuitively seen that the specific heat capacity (Δ*C*_P_) of high-performance cold-mix resin and high-performance cold-mix resin asphalts exhibit a sudden change between −20 °C and 40 °C and the corresponding glass transition temperature are both about 12.4 °C. The low glass transition temperature indicates that high-performance cold-mix resin and high-performance cold-mix resin asphalts in low temperature conditions exhibit good deformation capacity. Generally, the glass transition temperature of pure asphalt is about −20 °C, which indicates that pure asphalt shows good viscoelasticity.

### 3.3. Microscopic Appearance

**Figure 6:**
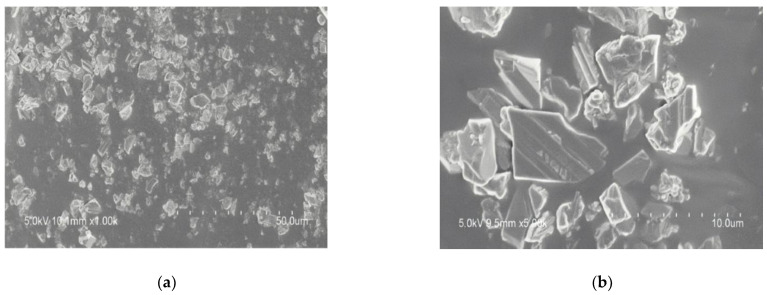
SEM images of cross-sections through high-performance cold-mix resin: (**a**) 1000 times; (**b**) 5000 times.

**Figure 7:**
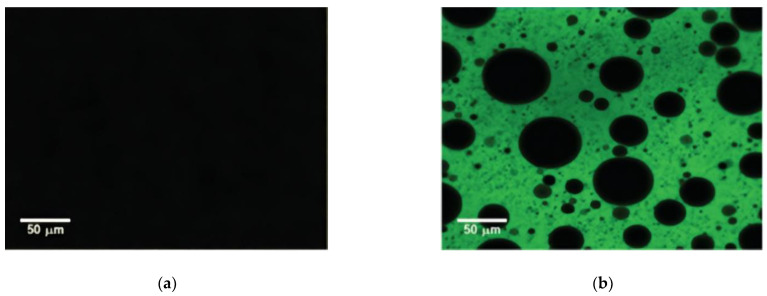
LSCM images: (**a**) asphalt; (**b**) high-performance cold-mix resin asphalts.

Figure 6 shows the SEM images of cross-sections through high-performance cold-mix resin. The phase-separated structure can be clearly seen from the brittle section of materials, which confers a beneficial toughness to the materials. Figure 7 shows the microscopic appearance of asphalt and high-performance cold-mix resin asphalts observed from LSCM. Under the irradiation of blue laser, the asphalt sample did not emit light and exhibited a black state, whereas the high-performance cold-mix resin in resin asphalt mixtures reflected fluorescence [36]. The resin asphalt mixtures contain two phases, one phase is high-performance cold-mix resin that forms the network structure skeleton, which is the main carrier for resin asphalt mixtures to exert strength; the other phase is pure asphalt distributed in the form of tiny particles in the network structure formed by high-performance cold-mix resin, which plays a role of filling and anti-corrosion. Due to the combined effect of the above two phases, high-performance cold-mix resin asphalts show good deformation capacity and toughness.

### 3.4. Influence of Component Content on Mechanical Properties of High-Performance Cold-Mix Resin

**Figure 8:**
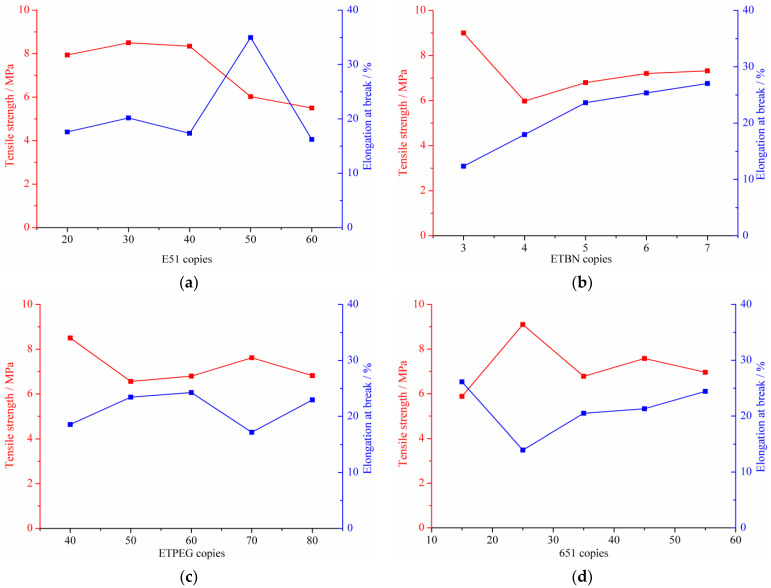

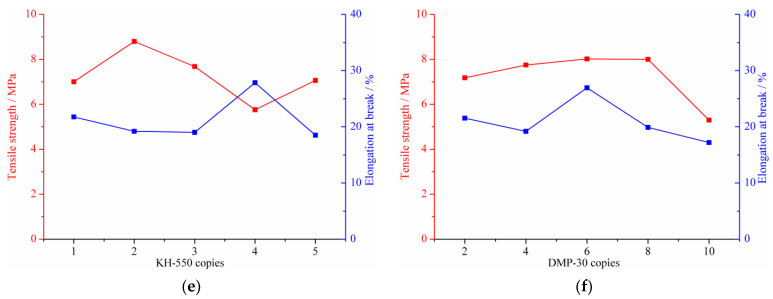
Tensile strength and elongation at break in dependence on the content of respective component: (**a**) E51; (**b**) ETBN; (**c**) ETPEG; (**d**) 651; (**e**) KH-550; (**f**) DMP-30.

The tensile strength as well as the elongation at break of high-performance cold-mix resin versus the content of respective component are depicted in Figure 8. With the increasing additive dosage of E51 epoxy resin, 651 polyamide curing agent and DMP-30 accelerator, the tensile strength of high-performance cold-mix resin first increases and then decreases. It is worth noting in Figure 8b,c that the addition of a small amount of flexibilizers ETBN and ETPEG can decrease the tensile strength of high-performance cold-mix resin, whereas with the increase of dosage, the tensile strength gradually increases. The KH-550 coupling agent exerts complex influences on the tensile strength and there is no obvious trend therein, but an extreme value is seen.

It also can be seen from the correlation between the mixing amounts of each composition and the elongation at break of high-performance cold-mix resin that, the flexibilizer ETBN can significantly increase the elongation at break, which almost shows a linear increase with additive dosage. The E51 resin, ETPEG, DMP-30 and KH-550 coupling agent all have an optimal concentration and an excessive dosage thereof will affect the toughness of high-performance cold-mix resin. With the increasing dosage of the 651 polyamide curing agent, the toughness and plasticity of high-performance cold-mix resin first decreases and then increases.

In general, it was found that six raw materials have certain effects on the tensile strength of high-performance cold-mix resin. On the basis of range analysis, it can be obtained that the epoxy resin matrix, ETBN, 651 polyamide curing agent and KH-550 coupling agent have relatively large range value and reach more than 3, indicating that the incorporation of the aforementioned four raw materials has a great impact on the tensile strength of high-performance cold-mix resin, whereas the range value for ETPEG diluter and DMP-30 accelerator is less than 3, demonstrating that the incorporation of these two raw materials exert relatively little influence on the tensile strength of high-performance cold-mix resin. In addition, based on the analysis of orthogonal tests, the range value in elongation at break for six compositions differ. The E51 epoxy resin has the largest influence on the toughness and the plasticity of high-performance cold-mix resin, whereas the generated influence of ETPEG is the smallest.

### 3.5. Pavement Performances

#### 3.5.1. Water Stability

Table 4 lists the test results for water stability of high-performance cold-mix resin mixtures and hot-mix epoxy asphalt mixtures. The residual Marshall stabilities of high-performance cold-mix resin mixtures and hot-mix epoxy asphalt mixtures were 97.4% and 98.0% whereas their freeze-thaw splitting tensile strength ratio were 90.0% and 98.9%, respectively. By comparison, it was found that the residual Marshall stability and freeze-thaw splitting tensile strength ratio of high-performance cold-mix resin mixtures is almost equivalent to those of hot-mix epoxy asphalt mixtures, whereas the Marshall stability and the splitting tensile strength of high-performance cold-mix resin mixtures are much larger than those of hot-mix epoxy asphalt mixtures.

#### 3.5.2. High and Low-Temperature Performances

Table 5 quantitatively shows the high and low-temperature performances of high-performance cold-mix resin mixtures and hot-mix epoxy asphalt mixtures. The high-performance cold-mix resin mixtures at 60 °C showed a dynamic stability 396 cycles/mm larger than that of hot-mix epoxy asphalt mixtures. This indicates that under high-speed traffic loads, the pavement with high-performance cold-mix resin mixtures as the paving material exhibited higher stability compared to the pavement with hot-mix epoxy asphalt mixtures as the paving material. Nevertheless, the road surface paved with the aforementioned two materials showed good stability and no rutting and other diseases emerged in the actual project. It also can be observed that the high-performance cold-mix resin mixtures showed approximately the same flexural tensile strength and ultimate flexural tensile strain as hot-mix epoxy asphalt mixtures. In fact, due to the high asphalt-aggregate ratio (7.5%), high-performance cold-mix resin mixtures are more rigid than hot-mix epoxy asphalt mixtures.

### 3.6. Fatigue Performance

The experimental data obtained from the fatigue tests are list in Table 6. Under the stress level of 400 με, the initial stiffness modulus of high-performance cold-mix resin mixtures reached 21,000 MPa in the four-point bending fatigue test, which is 30% higher than that of hot-mix epoxy asphalt mixtures. Therefore, the fatigue life of the high-performance cold-mix resin mixtures was much greater than that of hot-mix epoxy asphalt mixtures, as shown in Table 6. In actual engineering, the applied high-performance cold-mix resin mixtures instead of hot-mix epoxy asphalt mixtures is conducive to reinforcing the steel plates, sharing load on bridge decks and reducing vertical displacement of steel plates, thus improving the fatigue life of steel plates.

## 4. Conclusions

The results obtained appear to support the following conclusions:

The results of IR spectrum analysis and comparison with the data from spectrum library show that the main compositions in components A of Dow resins, TY-RA resins and YC-YN resins are DER332, DER 324 and EPON 828, respectively. Component B in Dow resins is Hardener XU-HY 943, mainly including (N, N) dimethyldipropylamines and polyamides, while that in TY-RA resins is the Paramul ERO-SB compound, mainly consisting of polyamides. Moreover, the room-temperature curing agent containing polyamides is mainly found in Component B of YC-YN resins.

Through DSC, different exothermic peaks appear at different heating rates of 5, 10, 15 and 20 °C/min after curing cold-mix epoxy asphalt mixtures and the faster the rate of heating, the higher the peak temperatures (about 90, 105, 115 and 125 °C, respectively). Both cold-mix resin and cold-mix resin asphalt show a sudden change in specific heat capacity (△*C*_P_) in the range of −20 °C to 40 °C and the corresponding glass transition temperature is about 12.4 °C, presenting a low *T*_g_, that is, a high denaturation capacity can be retained even at this low temperature.

The results of orthogonal fracture tensile test and range analysis illustrate that the effect of resin matrix, ETBN, 651 polyamide curing agent and KH-550 coupling agent content on tensile strength of high-performance cold-mix resin is greater than that of ETPEG diluter and DMP-30 accelerator content on tensile strength of high-performance cold-mix resin. In terms of the elongation at break of high-performance cold-mix resin, the dosage of E51 resin generates the greatest effect whereas the ETPEG generates the least effect.

Pavement performance tests show that high-performance cold-mix resin mixtures exhibits comparable residual Marshall stability and freeze-thaw splitting tensile strength ratio as well as flexural tensile strength and ultimate flexural tensile strain at low temperature, whereas larger Marshall stability and splitting tensile strength as well as greater dynamic stability at high temperature, compared with hot-mix epoxy asphalt mixtures. The four-point bending tests show that, at strain of 400 με, the fatigue life of high-performance cold-mix resin mixtures is much greater than that of hot-mix epoxy asphalt mixtures.

## Figures and Tables

**Table 1:** The grading envelope and composite gradation of high-performance cold-mix resin mixtures.

Sieve Size (mm)	Mass Percentage of the Mixtures Passing through the Following Sieves (%)									
	16.0	13.2	9.5	4.75	2.36	1.18	0.6	0.3	0.15	0.075
Upper limit (%)	100	100	85	68	50	38	28	20	15	12
Lower limit (%)	100	90	68	38	24	15	10	7	5	4
Composite gradation	100.0	97.1	78.5	46.3	38.5	30.5	19.5	13.4	11.1	9.5

**Table 2:** The grading envelope and composite gradation of hot-mix epoxy asphalt mixtures.

Sieve Size (mm)	Mass Percentage of the Mixtures Passing through the Following Sieves (%)									
	16.0	13.2	9.5	4.75	2.36	1.18	0.6	0.3	0.15	0.075
Upper limit (%)	100	100	100	85	70	55	40	32	23	14
Lower limit (%)	100	100	95	65	50	39	28	21	14	7
Composite gradation	100.0	100	97.5	75	60	45.5	34	25	17	10.5

**Table 3:** Results of IR spectrum analysis of three typical epoxy resins.

Items	Component A	Component B
DOW epoxy resin	DER 332	Hardener XU-HY 943 *
TY-RA epoxy resin	DER 324	Paramul ERO-SB ***
YC-YN epoxy resin	EPON 828	Paramul ERO-SB

Notes: Hardener XU-HY 943 * includes (N, N) dimethyldipropylamines and polyamides as efficient curing agent and Paramul ERO-SB *** comprises polyamide (50~70%), mineral oil (10~30%), 2-butoxyethanol (5~10%) and 2-(2-butoxyethoxy) ethanol (5~10%).

**Table 4:** Test results for water stability of high-performance cold-mix resin mixtures and hot-mix epoxy asphalt mixtures.

Index	Test Result	
	High-Performance Cold-Mix Resin Mixtures	Hot-Mix Epoxy Asphalt Mixtures
Immersed Marshall stability (kN)	77.91	56.06
Standard Marshall stability (kN)	80.00	57.20
Residual Marshall stability MS_0_ (%)	97.4	98.0
Splitting tensile strength after freeze-thaw cycles (MPa)	7.21	2.79
Standard splitting tensile strength (MPa)	8.02	2.82
Freeze-thaw splitting tensile strength ratio TSR (%)	90.0	98.9

**Table 5:** Temperature performances of high-performance cold-mix resin mixtures and hot-mix epoxy asphalt mixtures.

Condition	Index	Test Result	
		High-Performance Cold-Mix Resin Mixtures	Hot-Mix Epoxy Asphalt Mixtures
High temperature (60 °C)	Dynamic stability (cycles/mm)	62,327	61,931
Low temperature (−10 °C)	Flexural tensile strength (MPa)	35.2	37.3
	Ultimate flexural tensile strain (με)	4158.0	4157.2

**Table 6:** Fatigue performance of high-performance cold-mix resin mixtures and hot-mix epoxy asphalt mixtures.

Stress Level (με)	Index	Test Result	
		High-Performance Cold-Mix Resin Mixtures	Hot-Mix Epoxy Asphalt Mixtures
400	Initial stiffness modulus (MPa)	21,000	16,200
	Mean fatigue life (×10^4^ cycles)	>100	20

## Data Availability

The data presented in this study are available on request from the correspondence author.

## References

[1] Siwowski T., Kulpa M., Janas L. (2019). Remaining fatigue life prediction of welded details in an orthotropic steel bridge deck. J. Bridge Eng..

[2] Wang F., Tian L.J., Lyu Z.D., Zhao Z., Chen Q.K., Mei H.L. (2021). Stability of full-scale orthotropic steel plates under axial and biased loading: Experimental and numerical studies. J. Constr. Steel Res..

[3] Xiao Z.G., Yamada K., Ya S., Zhao X.L. (2008). Stress analyses and fatigue evaluation of rib-to-deck joints in steel orthotropic decks. Int. J. Fatigue.

[4] Chen X.H., Huang W., Qian Z.D., Zhang L. (2017). Design principle of deck pavements for long-span steel bridges with heavy-duty traffic in China. Road. Mater. Pavement..

[5] Kolstein M.H. (2007). Fatigue Classification of Welded Joints in Orthotropic Steel Bridge Decks. Ph.D. Thesis.

[6] Corte W.D., Boel V., Helincks P., Schutter G.D. (2016). Fatigue assessment of a lightweight steel-concrete bridge deck concept. Bridge Struct..

[7] Wang C.S., Feng Y.C., Duan L. (2009). Fatigue damage evaluation and retrofit of steel orthotropic bridge decks. Key Engineering Materials.

[8] Cui C., Xu Y.L., Zhang Q.H. (2021). Multiscale fatigue damage evolution in orthotropic steel deck of cable-stayed bridges. Eng. Struct..

[9] Fang Z., Ding Y.L., Wei X.C., Li A.Q., Geng F.F. (2020). Fatigue failure and optimization of double-sided weld in orthotropic steel bridge decks. Eng. Fail. Anal..

[10] Martinho F.C.G., Picado-Santos L.G., Capitao S.D. (2018). Influence of recycled concrete and steel slag aggregates on warm-mix asphalt properties. Constr. Build. Mater..

[11] Kim Y.J., Im S.H., Lee D., Hwang S.D. (2008). Evaluation of warm mix asphalt mixtures with foaming technology and addtives using new simple performance testing equipment. Int. J. Highway Eng..

[12] Pan R., Li Y.M. (2020). Effect of warm mix rubber modified asphalt mixture as stress absorbing layer on anti-crack performance in cold region. Constr. Build. Mater..

[13] Zeng G.D., Xu W., Huang H.M., Zhang X.N. (2019). Study on the microstructure and properties of hot-mix epoxy asphalt. Int. J. Pavement Res. Technol..

[14] Cristescu N.D., Craciun E.M., Soós E. (2004). Mechanics of Elastic Composites.

[15] Xu W., Wei J.T., Chen Z.X., Wang F., Zhao J. (2021). Evaluation of the Effects of Filler Fineness on the Properties of an Epoxy Asphalt Mixture. Materials.

[16] Fuhaid A.A., Lu Q., Luo S. (2018). Laboratory Evaluation of Biobased Epoxy Asphalt Binder for Asphalt Pavement. J. Mater. Civ. Eng..

[17] Min Z.H., Wang Q.C., Xie Y.X., Xie J.Q., Zhang B. (2020). Influence of polyethylene glycol chain on the performance of epoxy asphalt binder and mixture. Constr. Build. Mater..

[18] Zhang Z.P., Sun J., Huang Z.G., Wang F., Jia M., Lv W.J., Ye J.J. (2021). A laboratory study of epoxy/polyurethane modified asphalt binders and mixtures suitable for flexible bridge deck pavement. Constr. Build. Mater..

[19] Gaul R.W. (1996). Epoxy asphaly concrete- a polymer concrete with 25 years’ experience. Am. Soc. Test. Mater..

[20] Yu J.Y., Cong P.L., Wu S.P. (2009). Laboratory investigation of the properties of asphalt modified with epoxy resin. J. Appl. Polym. Sci..

[21] Kang Y., Song M.Y., Pu L., Liu T.F. (2015). Rheological behaviors of epoxy asphalt binder in comparison of base asphalt binder and SBS modified asphalt binder. Constr. Build. Mater..

[22] Ahmedzade P., Yilmaz M. (2008). Effect of polyester resin additive on the properties of asphalt binders and mixtures. Constr. Build. Mater..

[23] Zhang Z., Li J.S., Wang Z.Y., Long S.Y., Jiang S.J., Liu G.L. (2020). Preparation and performance characterization of a novel high-performance epoxy resin modified reactive liquid asphalt. Constr. Build. Mater..

[24] Liu Y., Qian Z.D., Shi X.J., Zhang Y.H., Ren H.S. (2021). Developing cold-mixed epoxy resin-based ultra-thin antiskid surface layer for steel bridge deck pavement. Constr. Build. Mater..

[25] Lei Y.B., Cao X.J. (2015). Preparation of epoxy-resin concrete using microwave curing method and its pavement performance evaluation. J. Mater. Civ. Eng..

[26] Ge Z.S., Wang H., Zhang Q.S., Xiong C.L. (2015). Glass fiber reinforced asphalt membrane for interlayer bonding between asphalt overlay and concrete pavement. Constr. Build. Mater..

[27] Shanbara H.K., Ruddock F., Atherton W. (2018). A laboratory study of high-performance cold mix asphalt mixtures reinforced with natural and synthetic fibres. Constr. Build. Mater..

[28] Ma S.Q., Liu W.Q., Hu C.H., Wang Z.F., Tang C.Y. (2010). Toughening of epoxy resin system using a novel dendritic polysiloxane. Macromol. Res..

[29] GB/T 6040-2002 (2002). General Rules for Infrared Analysis.

[30] Hanaoka T., Arao Y., Kayaki Y., Kuwata S., Kubouchi M. (2021). Analysis of nitric acid decomposition of epoxy resin network structures for chemical recycling. Polym. Degrad. Stabil..

[31] Zhang H., Gao P.W., Pan Y.Q., Li K., Zhang Z.X., Geng F. (2020). Development of cold-mix high-toughness resin and experimental research into its performance in a steel deck pavement. Constr. Build. Mater..

[32] Si J.J., Jia Z.X., Wang J.Y., Yu X., Li Y., Dong F.Q., Jiang R.L. (2018). Comparative analysis of cold-mixed epoxy and epoxy SBS-modified asphalts: Curing rheology, thermal, and mechanical properties. Constr. Build. Mater..

[33] JTG E20-2011 (2011). Standard Test Methods of Bitumen and Bituminous Mixtures for Highway Engineering.

[34] Han S.O., Drzal L.T. (2003). Water absorption effects on hydrophilic polymer matrix of carboxyl functionalized glucose resin and epoxy resin. Eur. Polym. J..

[35] Liu J.Y., Sun Y.R., Wang W.Y., Chen J.Y. (2017). Using the viscoelastic parameters to estimate the glass transition temperature of asphalt binders. Constr. Build. Mater..

[36] Kang Y., Chen Z.M., Jiao Z., Huang W. (2010). Rubber-like thermosetting epoxy asphalt composites exhibiting atypical yielding behaviors. J. Appl. Polym. Sci..

